# Er:YAG laser irradiation enhances bacterial and lipopolysaccharide clearance and human gingival fibroblast adhesion on titanium discs

**DOI:** 10.1038/s41598-021-03434-1

**Published:** 2021-12-14

**Authors:** Chen-Ying Wang, Bor-Shiunn Lee, Ya-Ting Jhang, Kevin Sheng-Kai Ma, Chen-Pang Huang, Kuan-Lun Fu, Chern-Hsiung Lai, Wan-Yu Tseng, Mark Yen-Ping Kuo, Yi-Wen Chen

**Affiliations:** 1 Department of Dentistry, National Taiwan University Hospital, Taipei, Taiwan; 2 School of Dentistry, National Taiwan University, Taipei, Taiwan; 3 Graduate Institute of Oral Biology, School of Dentistry, National Taiwan University, Taipei, Taiwan; 4 Graduate Institute of Clinical Dentistry, School of Dentistry, National Taiwan University and National Taiwan University Hospital, No. 1 Chang-Te Street, Taipei, 10048 Taiwan; 5 Graduate Institute of Biomedical Electronics and Bioinformatics, College of Electrical Engineering and Computer Science, National Taiwan University, Taipei, Taiwan; 6 College of Life Science, Kaohsiung Medical University, Kaohsiung, Taiwan

**Keywords:** Microbiology, Materials science

## Abstract

To investigate the effect of Er:YAG laser treatment on lipopolysaccharide (LPS) clearance and fibroblast adhesion on titanium disks. Grade IV titanium discs (n = 216) were used and allocated to 6 groups. Group 1 was the negative control without *Porphyromonas gingivalis* inoculation. Discs in Groups 2–6 were incubated with *P. gingivalis* to form a biofilm. Group 3 received 0.12% chlorhexidine irrigation and Group 4 received titanium curettage to remove the biofilm. Group 5 was treated with Er:YAG laser irradiation and Group 6 was treated with titanium curettage plus Er:YAG laser irradiation. The contact angle and surface roughness were measured after the various treatments. The surface microstructure and residual bacteria were examined using scanning electron microscopy and confocal laser scanning microscopy, respectively. Residual LPS was examined using a limulus amoebocyte lysate assay and human gingival fibroblast adhesion was quantified using fluorescent microscopy. Curettage plus Er:YAG laser irradiation was the most effective method for removing bacteria and LPS. No significant difference in the amount of fibroblast adhesion was found between the control and Group 6. Combined use of Er:YAG laser irradiation and curettage optimizes LPS clearance and fibroblast adhesion on titanium discs.

## Introduction

Peri-implantitis is characterized by progressive loss of supporting bone and inflammation of peri-implant connective tissue^1^. The patient- and implant-level prevalence of peri-implantitis has been reported to be approximately 22–45% and 11.4%, respectively^2–4^. Peri-implantitis is a multifactorial disorder that may be initiated by iatrogenic factors such as surgical technical complications, implant mal-positioning, inadequate restoration-abutments seating, over-contoured restorations, and excess cement remnants^5^. The other etiology of peri-implantitis involves bacterial biofilm accumulation^1,6,7^, in which pathogenic species including *Porphyromonas gingivalis*^8^ and virulence factors such as lipopolysaccharides (LPS)^9^ have been identified. The structure difference in vascularity or connective fibers between peri-implant and periodontal tissues may influence the host response to biofilms accumulation^10^. Therefore, peri-implantitis shows rapid progress than periodontitis.

The primary cause for the onset of infection is a critical factor for successful treatment of peri-implantitis. For peri-implantitis whose etiology is bacterial origin, the primary treatment goal of peri-implantitis is to remove the biofilm and LPS from implant surfaces to resolve soft tissue inflammation and prevent further bone loss. Mechanical, non-surgical therapy for peri-implantitis lesions remains unpredictable, with clinical benefits limited to 6–12 months^11^, and can alter the microstructure of the implant surface^12^. An alternative treatment for peri-implantitis is laser therapy. Among various minimally invasive lasers^13–15^, the erbium-doped yttrium aluminum garnet (Er:YAG) laser has been frequently employed for peri-implantitis management^13–16^. In addition to its ability to ablate hard tissue^17,18^, the wavelength of the Er:YAG laser is approximate to the absorption wavelength of LPS, which enables the Er:YAG laser to exert a bactericidal effect on implant surfaces^19–24^. Moreover, Er:YAG laser exhibits strong affinity to water which causes microexplosions and achieves sterilization. The biofilm is composed of bacteria and an aqueous mucopolysaccharide matrix that can be effectively eliminated by the Er:YAG laser with a water spray^25,26^. Er:YAG laser treatment of titanium implants creates a surface with appropriate wettability^20,27^ that favors the adhesion of epithelial cells, gingival fibroblasts, and osteoblast-like cells^23^. Furthermore, Er:YAG laser irradiation is effective for the debridement and removal of inflamed granulomatous tissue from peri-implant defects^1^.

Previous in vitro studies have demonstrated the efficacy of Er:YAG laser treatment in the ablation of bacterial biofilms or calculus^23,28^. However, residual bacteria and LPS after Er:YAG laser ablation might impede the attachment of human gingival fibroblasts (HGFs) and this topic has not been studied. We conducted this study to address the effect of Er:YAG laser treatment on residual bacteria and LPS in an in vitro titanium-based peri-implantitis model.

## Results

### Wettability measurement of the titanium surface

**Figure 1:**
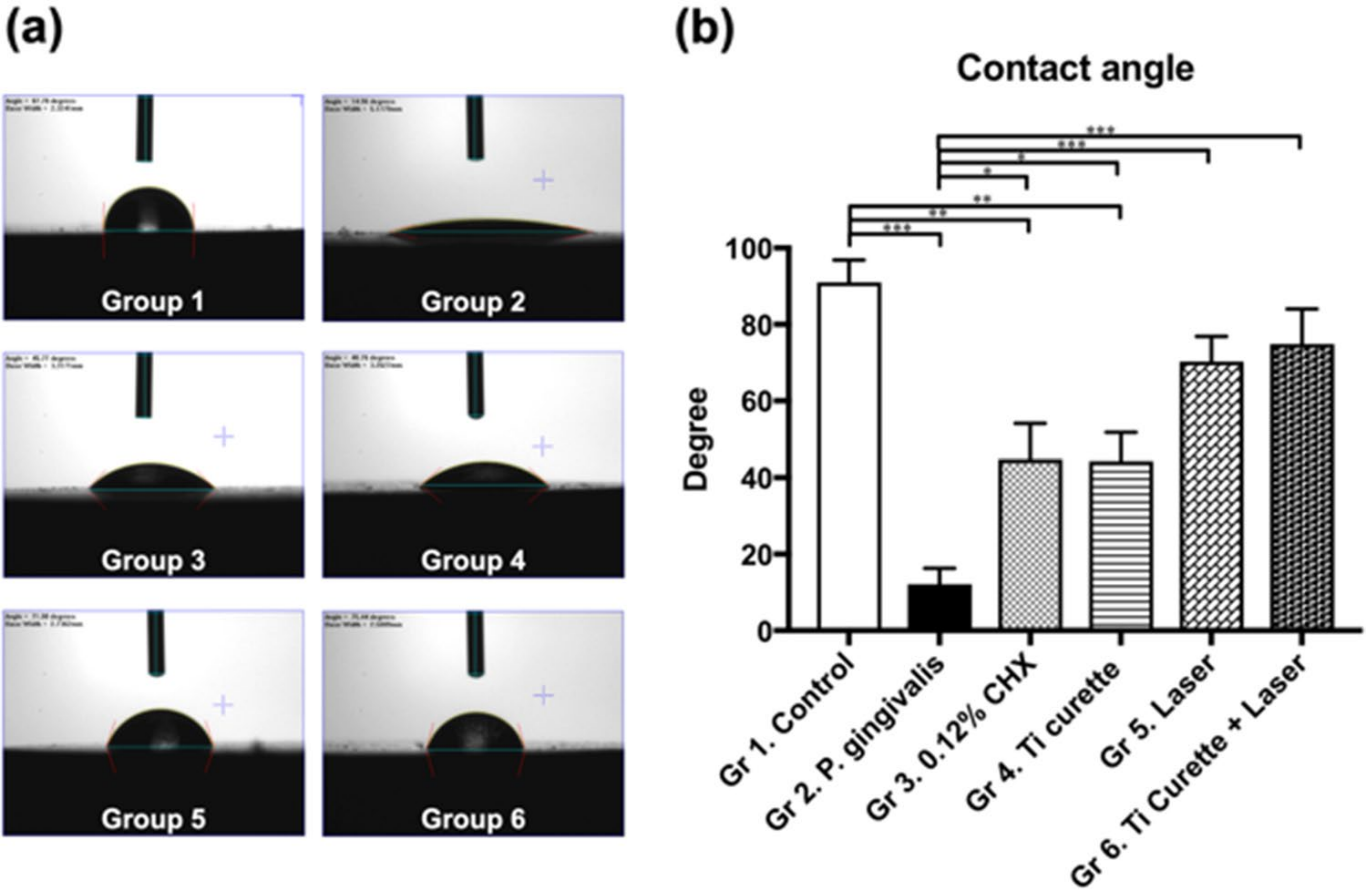
(**a**) Contact angle photos of *P. gingivalis* biofilm inoculation on titanium discs after different treatments. (**b**) Contact angle of *P. gingivalis* biofilms after different treatment. *p < 0.05 **p < 0.01 ***p < 0.001.

The contact angle of pure titanium plates (Group 1) was significantly higher than those of titanium discs with the *P. gingivalis* biofilm, 0.12% CHX washing, and curettage (*p* < 0.0001, *p* < 0.001, *p* < 0.05). For Er:YAG laser treatment alone, or the combined use of curettage and Er:YAG laser treatment, the contact angle was not significantly different from that of the control group (Fig. 1).

### Surface roughness analysis and measurement

**Figure 2:**
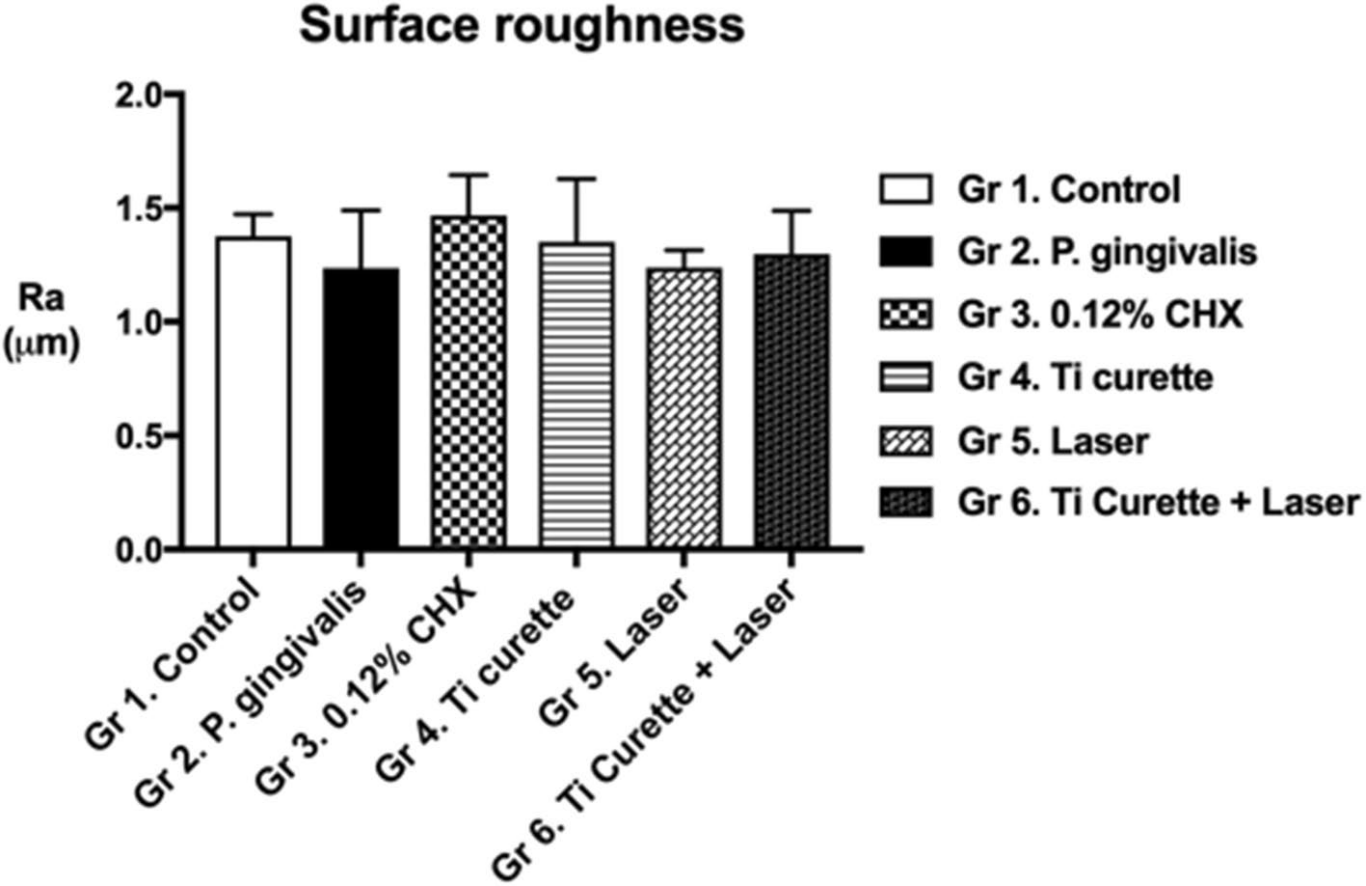
Surface roughness analysis (Ra) of *P. gingivalis* inoculation on titanium discs after different treatments. *p < 0.05 **p < 0.01 ***p < 0.001.

Surface roughness indicated the influence of the biofilm, CHX washing, curettage, laser treatment, and the combination of laser treatment and curettage on the surface structure of the titanium plates. No significant differences were found among the groups (Fig. 2). These findings suggested that neither mechanical debridement nor Er:YAG irradiation altered Ra.

### SEM findings of the titanium surface following debridement

**Figure 3:**
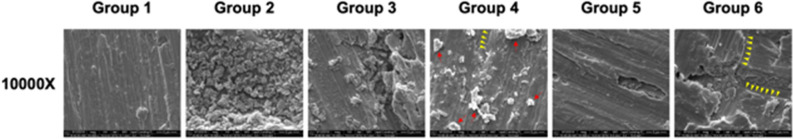
Scanning electron microscope images of titanium discs after various treatments (Group 1–Group 6) at magnifications of × 10,000. The red arrow represents titanium particles. The yellow arrow represents scratches caused by curettage.

No bacteria were observed on the titanium surface in the control group (Fig. 3). By contrast, a *P. gingivalis* biofilm was prominently observed in Group 2. After 0.12% CHX washing (Group 3), *P. gingivalis* clusters were still visible on the titanium surface. Similarly, titanium debris embedded with bacterial residue was observed after curettage plus PBS washing (Group 4). These findings indicated that curettage alone was insufficient to remove all bacteria. By contrast, Er:YAG laser debridement (Group 5) was effective at removing *P. gingivalis* and the surface clearance was similar to that of the control group. Curettage followed by Er:YAG laser treatment (Group 6) was also effective at removing *P. gingivalis*. However, horizontal and vertical scratches caused by curettage were observed.

### Live and dead bacteria on the titanium surface under confocal microscopy

**Figure 4:**
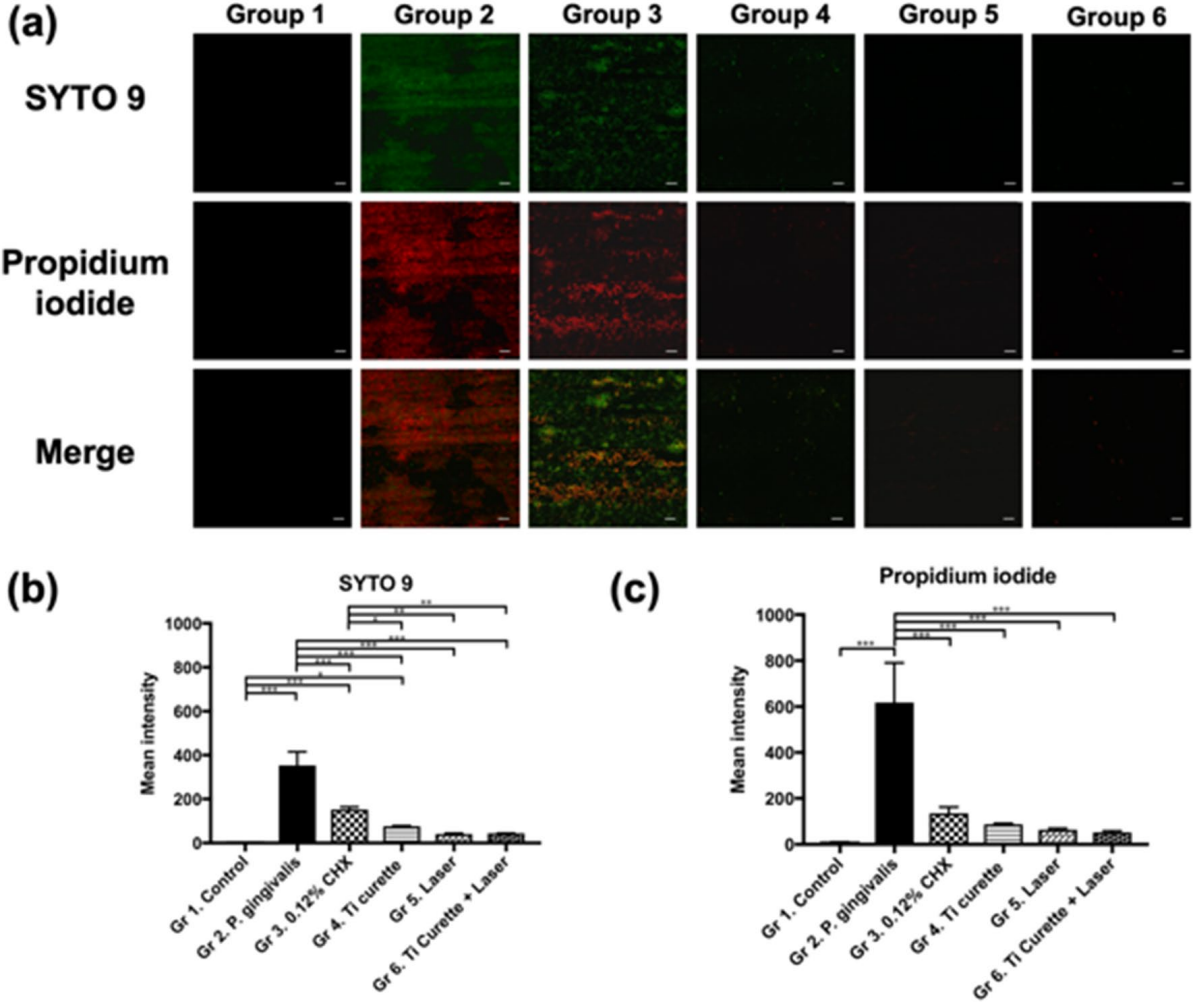
(**a**) Representative fluorescence microscopy images (× 63) with live (green; SYTO9)/dead (red; propidium iodide) staining of *P. gingivalis* ATCC 33277 adhesion on rough titanium disc surfaces after different treatments. Each image was taken with the same × 63 object lens with oil and z-stacked for comparison. Scale bar, 10 μm (white). (**b**) Mean intensity of SYTO 9 and (**c**) propidium iodide after various treatments. *p < 0.05 **p < 0.01 ***p < 0.001.

SYTO 9 and propidium iodide were used to distinguish live and dead bacteria based on their different abilities to penetrate the cell membrane (Fig. 4a). The control group (Group 1) did not produce fluorescent signals because it did not contain bacteria. The fluorescence intensity of *P. gingivalis* in Group 2 was the highest among the 6 groups. Bacterial counts decreased after 0.12% CHX washing (Group 3) but bacteria were not completely removed. Curettage (Group 4) eliminated most bacteria, resulting in fewer fluorescent signals compared with Group 3. Laser irradiation (Group 5) and combined use of curettage and laser irradiation (Group 6) resulted in even fewer fluorescent signals, with only a few scattered red signals being observed. Quantitative analysis of fluorescence intensity using Zen software revealed that Group 2 had the highest fluorescence intensity (Fig. 4b, c), followed by Group 3 (0.12% CHX washing) and Group 4 (curettage). Groups 5 and 6 had the lowest fluorescence intensity and these results were consistent with the SEM findings.

### Kinetic turbidimetric assay for residual endotoxins

**Figure 5:**
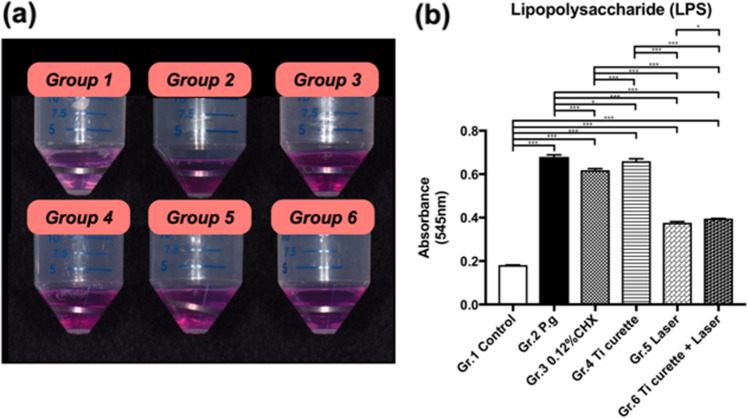
(**a**) Photos of residual LPS observed during the LAL assay of the titanium discs following different surface treatments. (**b**) Ultraviolet (UV) absorbance of the residual LPS on titanium discs following various treatments using UV spectrum absorbance at 545 nm. *p < 0.05 **p < 0.01 ***p < 0.001.

After immersion of the titanium discs in endotoxin-free LAL water for 24 h, the absorbance in the kinetic turbidimetric assay for titanium discs was measured (Fig. 5). The absorbance of titanium discs with *P. gingivalis* biofilm formation (Group 2) was 0.679 ± 0.009, which was the highest among the 6 groups. After 0.12% CHX washing (Group 3), the absorbance was significantly lower than that of Group 2 (*p* < 0.05). The absorbance after curettage (Group 4) was not significantly different from that of Group 2 (*p* = 0.078). After Er:YAG laser irradiation and after combined use of curettage and Er:YAG laser irradiation, the absorbance was significantly lower than that of Group 2 (*p* < 0.0001, *p* < 0.0001, respectively). These findings suggest that Er:YAG laser treatment was able to reduce residual endotoxins on the titanium surface, such as LPS.

### Quantitative evaluation of HGF adhesion after different surface treatments

**Figure 6:**
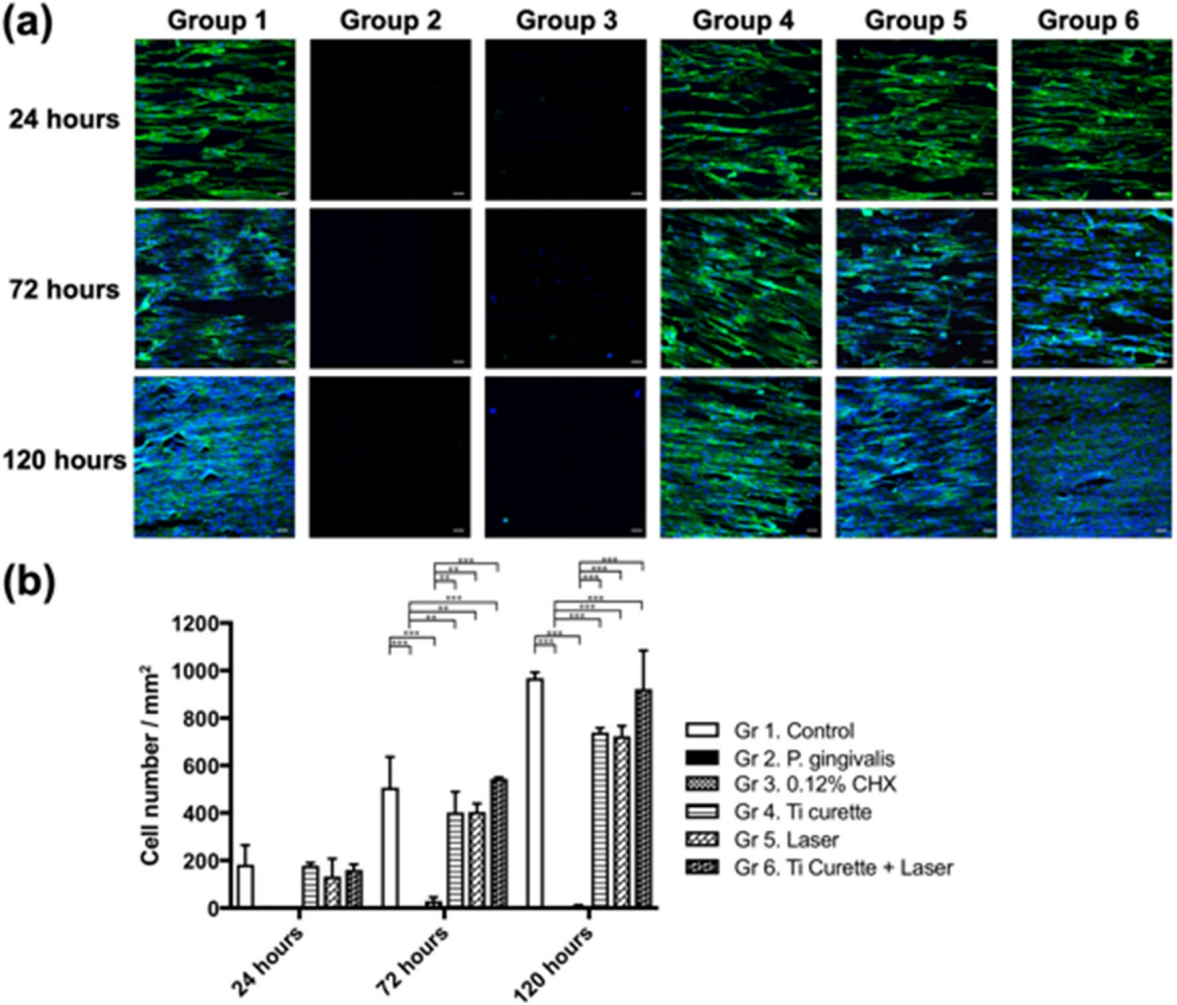
(**a**) Representative fluorescence microscopy images (× 20) of HGF cells stained with ActinGreen^Tm^ 488 (green) indicate a cytoskeleton, and DAPI (blue) was used to label nucleic acids. Each image was taken with the same × 20 object lens and z-stacked for comparison. Scale bar, 50 μm (white). (**b**) HGF cell adhesion assay (cell number per mm^2^). The cell adhesion number of contaminated titanium discs after different treatments at 24, 72, and 150 h. *p < 0.05 **p < 0.01 ***p < 0.001.

Evaluation of HGF adhesion (per square millimeter) after different surface treatments was quantified using fluorescent microscopy. The results showed that the HGF adhesion in Group 2 was the lowest among the 6 groups (Fig. 6). In addition, combined use of curettage and Er:YAG laser irradiation (Group 6) resulted in the highest HGF adhesion, followed by curettage (Group 4) or Er:YAG laser treatment (Group 5) alone. The titanium discs treated using 0.12% CHX washing (Group 3) had the most unfavorable surface in terms of HGF adhesion.

## Discussion

This study investigated HGF adhesion to *P. gingivalis* biofilm-contaminated titanium discs that were treated with different clinically used methods for peri-implantitis, including CHX washing, mechanical debridement, and Er:YAG laser irradiation. The findings indicated that combined use of curettage and Er:YAG laser irradiation was the most effective at removing bacteria and residual endotoxins, and achieved a beneficial microenvironment for HGF attachment to the titanium surface.

The surface roughness and contact angle of a titanium plate after a designated treatment has been shown to be associated with cell adhesion^29–31^. In this study, SEM revealed that mechanical debridement using curettage created scratches on the titanium surface (Fig. 3), although the roughness was not significantly changed (Fig. 2). Regarding water-surface interactions, a large water contact angle indicates hydrophobicity, whereas a small water contact angle suggests hydrophilicity^32–34^. Moreover, contact angles of less than 10° are considered super hydrophilic, those between 10° and 90° are considered hydrophilic, those between 90° and 120° are considered hydrophobic, and those over 120° are considered superhydrophobic^32–34^. The contact angles of all groups in this study were less than 90°, indicating that the surfaces were hydrophilic. Specifically, the contact angles of Groups 2 to 6 were all significantly less than that of Group 1, which suggested that inoculation with bacteria and treatment including 0.12% CHX washing, titanium curettage, and laser treatment all altered surface hydrophilicity. The results may be attributable to residual endotoxins, such as LPS, and *P. gingivalis* on the titanium plates rendering the surfaces hydrophilic^35,36^. In addition, the contact angles of both Group 5 (Er:YAG laser treatment) and Group 6 (combined use of curettage and Er:YAG laser treatment) were higher than those of Groups 2–4. These findings were consistent with Group 6 demonstrating the most effective removal of bacteria and LPS (Figs. 4 and 5), causing similar HGF cell attachment to that of Group 1.

Group 2 exhibited the highest fluorescence intensity under confocal microscopy (Fig. 4) and SEM examination also showed a *P. gingivalis* colony 2–3 μm in size. Therefore, no HGF adhesion was observed at 24, 72, and 150 h, suggesting that *P. gingivalis* had an adverse effect on HGF cell adhesion and proliferation. This adverse bacterial effect on cell adhesion has been suggested to result from alteration of the extracellular matrix and its components^37–40^. Propidium iodide signals, which represented dead bacteria, were more intense than SYTO 9 signals, which represented live bacteria in all groups (Fig. 4). Residual bacterial contamination was more evident for 0.12% CHX treatment, as evidenced by higher fluorescence intensity, compared with mechanical debridement via titanium curettage, indicating that the latter was more effective at removing bacteria. In addition, Group 3 displayed a low density of nuclei characterized by DAPI, without any cytoskeletal signal under fluorescent microscopy (Fig. 6). Both the low cell count and the lack of cytoskeletal signals suggested poor HGF adhesion, which is in line with previous studies^41^. The reason was ascribed to the limited bactericidal effect of 0.12% CHX and the toxicity of 0.12% CHX to HGF. A previous in vitro study showed that CHX causes bacterial lysis by destroying the cell walls and inhibiting fibroblast attachment^42^. The bactericidal effect of Er:YAG laser irradiation combined with titanium curettage (Group 6) was superior to curettage alone (Group 4). The findings are in accordance with a previous study demonstrating that the bactericidal effect of Er:YAG laser irradiation on *P. gingivalis* is stronger than that of titanium curettage^23^. The amount of HGF attachment in the Ti curettage group was also lower than that of the Er:YAG laser irradiation group. The reason was ascribed to the greater amount of residual LPS after curettage than after laser treatment. The other possible reason was that curettage created debris on the surface of the titanium discs. The fluorescence signal intensity and LPS removal ability did not differ significantly between Er:YAG laser treatment with (Group 6) and without (Group 5) curettage. Nevertheless, Group 6 exhibited more HGF attachment than Group 5 and did not differ significantly from the control group. Previous studies have elucidated the utility of Er:YAG laser treatment in the clearance of bacterial biofilms ^23,28^, but the influence of residual bacteria and LPS after Er:YAG laser ablation on the attachment of HGFs has not been studied. LPS is released from the cell walls of gram-negative bacteria and can cause inflammation and toxicity to the host^9^. HGF attachment plays a critical role in the successful integration of gingival soft tissue and titanium implants^43^. The removal of LPS and HGF attachment achieved by laser irradiation plus curettage indicated the potential clinical efficacy for gingival fiber attachment to implant surfaces, which is crucial for forming a barrier against exposure to periodontal pathogens^44,45^.

This study used Grade 4 titanium plates (Ra = 1.3 μm) but the roughness of commercialized implants is approximately 1–3 μm, and the implant macro design, surface morphology, thread geometry, and thread pitch are more complicated, which has been shown to affect osseointegration^46^. Different roughnesses and complicated surface structures can influence the efficiency of surface treatments for implants^47,48^. In addition, peri-implantitis is typically accompanied by intrabony defects. The field of view is limited in clinical practice compared to in vitro models; both curettage and laser treatment are more feasible for achieving optimized bactericidal activity in an in vitro study. Furthermore, peri-implantitis is associated with complex bacterial species, and *P. gingivalis* is only one of the predominant bacteria^1^. In this study, we inoculated *P. gingivalis* on titanium discs, which cannot simulate complex biofilms on an implant surface in a true pocket. In addition, the implant topography and thread pattern are much more complex than the flat titanium discs used in this study. These are all limitations in determining the actual efficacy of Er:YAG laser with curettage for treating peri-implantitis.

In summary, this study was conducted with the aim of providing an optimal strategy for peri-implantitis management. The findings support the use of Er:YAG laser treatment with curettage to reduce bacteria and residual endotoxins, and optimize HGF attachment to implants, thereby achieving a beneficial peri-implant microenvironment for implant maintenance. Future studies could focus on identifying osteoblast adhesion on laser-treated titanium plates, as well as the osteogenic factors that underlie the process. The effect of laser treatment on the microenvironment of periodontium with peri-implantitis also requires further study.

## Methods

### Preparation of titanium samples

Titanium discs were prepared as described previously^49^. Briefly, 216 commercially pure Grade 4 titanium discs (ø = 15 mm, thickness: 2 mm, surface roughness: Ra = 1.3 µm; Ultimate Materials Technology, Hsinchu, Taiwan) were used. After being washed with distilled water and acetone 3 times in a Transsonic ultrasonic bath (Elma Ultrasonic, Singen, Germany), the titanium discs were autoclaved (121 °C, 2 atm, 30 min) and dried using laminar flow with UV irradiation overnight.

### P. gingivalis inoculation and surface treatments of the titanium discs

The bacterial inoculation was performed as follows. *P. gingivalis* (ATCC® 33277™) were cultured in a brain heart infusion broth (BD Bacto, REF 237500) supplemented with yeast extract, L-cysteine hydrochloride, and resazurin. Subsequently, *P. gingivalis* with optical density = 0.1 at 600 nm was inoculated on the titanium discs and then placed in a 24-well plate and cultured under anaerobic conditions for 72 h to allow biofilm formation.

**Figure 7:**
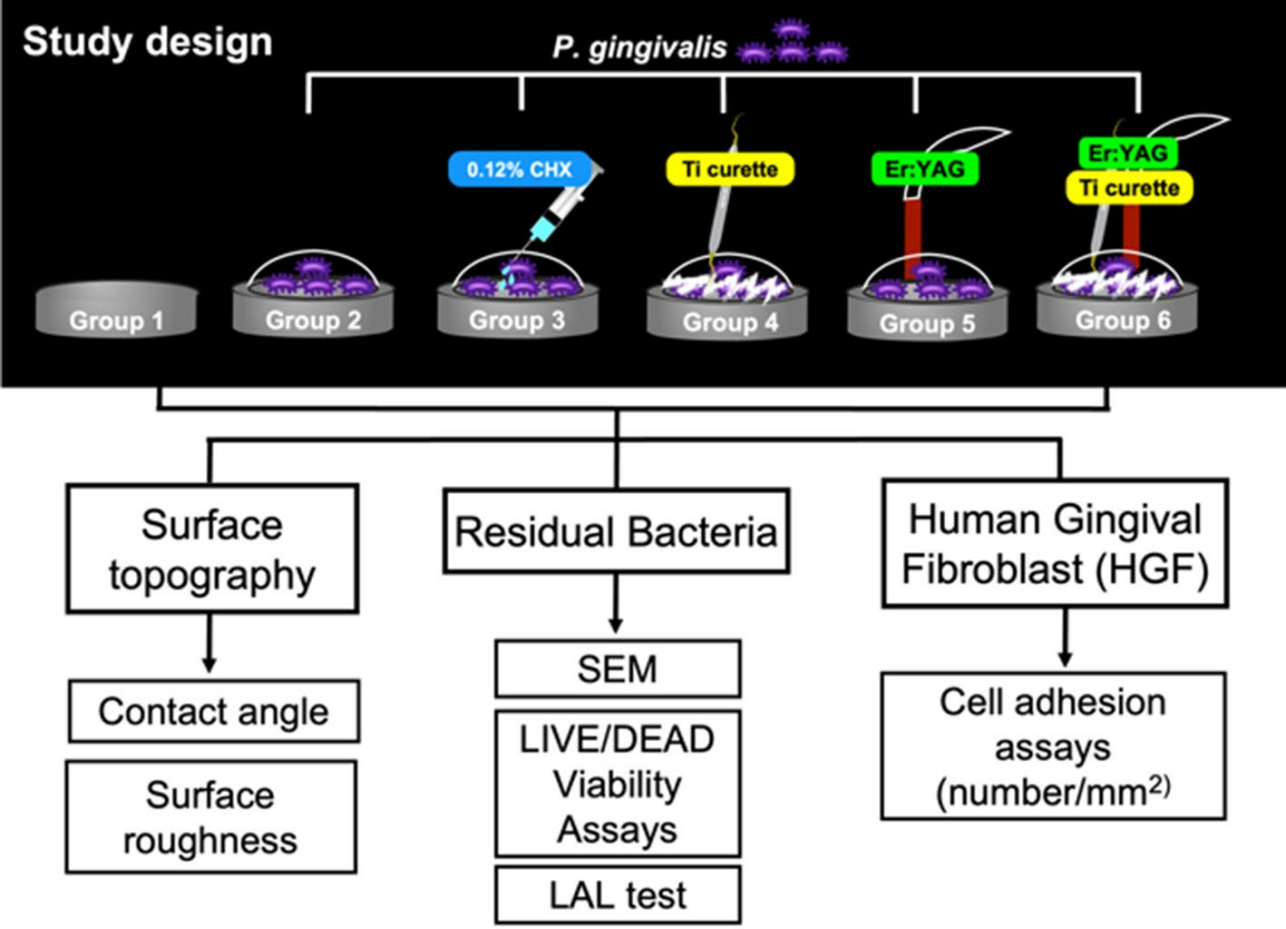
The study design consisted of 3 parts, including *P. gingivalis* bacterial assessment, an HGF adhesion test, and surface topography analysis. Group 1: negative control; Group 2–Group 6: *P. gingivalis* adhesion on titanium discs; Group 2: positive control without treatment; Group 3: treatment with 0.12% CHX; Group 4: mechanical debridement with Ti curettage; Group 5: treatment with Er:YAG laser irradiation; Group 6: combined debridement with Ti curettage and Er-YAG laser irradiation.

The sterilized titanium discs were divided into 6 groups (Fig. 7). Group 1 did not receive bacterial inoculation or debridement and served as a control. Group 2 received only *P. gingivalis* inoculation without further treatment. Group 3 received *P. gingivalis* inoculation followed by 0.12% chlorhexidine (CHX) irrigation (10 mL each time, 30 mL in total). Group 4 received *P. gingivalis* inoculation followed by periodontal titanium curette (8 mm in diameter, Langer 1/2, item code: 7103, Kohler Medizintechnik, GmbH & Co, Ltd, Stockach, Germany) debridement (30 vertical strokes with hand pressure for each disc) and normal saline irrigation (5 times per 10 strokes, 1 mL each time). Group 5 received *P. gingivalis* inoculation followed by Er:YAG laser (AdvErL Evo, Morita Corporation, Tokyo, Japan) irradiation using a C600F tip (600 μm in diameter, item code: no. 34-8001703, Morita Corporation) with water spray (panel settings for air and water flow were 1.49 L/min and 2.75 mL/min; 27.5 mL of water was used for 10 min). Group 6 received *P. gingivalis* inoculation followed by curette debridement and Er:YAG laser irradiation (combining the treatments of Groups 4 and 5). The calibration and standardization of the tip output was performed using a power meter (FieldMaster™ and LM-P10i, Coherent, Santa Clara, CA, USA) before laser irradiation. The settings were 80 mJ/pulse, 25 pulse per second, the tip transmittance was 62%, the distance from the tip end was 1 mm, the beam divergence was 5.2°, the pulse duration (pulse width) was 300 μs, the radiant exposure was 10.33 J/cm^2^ according to the formula provided by Morita Corporation. The radiant exposure rate of decline was 0.58 at 1-mm irradiation distance. Taking the titanium surface (176.625 mm^2^) and irradiation time (600 s) into consideration, the total radiant exposure was 245.7 J/cm^2^ (please see the official formula provided by Morita Corporation in supplementary data). The speed of the sweeping motion was approximately 0.5 mm/s in the left to right and top to bottom directions to cover the whole titanium disc surface. The laser treatment was performed by a well-trained periodontist (YT Jhang). Intra-examiner agreement was calculated with an intraclass correlation coefficient and agreement was 0.92 (95% CI: 0.88–0.95).

### Contact angle measurement (n = 6 for each group)

The surface hydrophilicity of the titanium discs after surface treatment was determined by measuring the contact angle (n = 6) with one drop (0.5 μL) of deionized water using a contact angle goniometer (FTA125; First Ten Ångstroms, Portsmouth, VA, USA). Three independent measurements were performed for each sample.

### Surface roughness measurement (n = 6 for each group)

To evaluate the surface roughness of the titanium discs, a Surfcorder ET 200 profilometer (Kosaka, Tokyo, Japan) was used (n = 6). The tracing diamond tip was 2 µm with a tracing length of 4 mm, force of 200 µN, tracing speed of 0.2 m/s, and cutoff value of 0.8 mm. Six tracings were performed at different locations on the surface of each specimen. The average surface roughnesses were calculated as Ra values.

### Surface morphology observation using scanning electron microscopy (n = 6 for each group)

The titanium discs (n = 6) were washed with phosphate-buffered saline (PBS) twice and dehydrated with serial ethanol (50%, 70%, 80%, 90%, 95%, 99%, for 15 min each) after surface treatments. After dehydration, the samples were dried using a critical point dryer, mounted on aluminum stubs, sputter-coated with approximately 20-nm-thick gold/palladium, and finally examined using field-emission scanning electron microscopy (FE-SEM; Nova NanoSEM 230, FEI Co, Brno, Czech Republic) with an accelerating voltage of 15 kV.

### Detection and quantification of adherent bacteria (n = 6 for each group)

The double staining of SYTO 9/PI has been used to quantify viable and dead bacteria. In the present study, adherent bacteria were examined and quantified using the SYTO 9/PI staining technique (LIVE/DEAD BacLight Bacterial Viability Kit; Invitrogen, Carlsbad, CA, USA). After fixation in 4% paraformaldehyde for 10 min, SYTO 9/PI double staining was performed on the titanium discs (n = 6) at 37 °C in the dark, followed by incubation for 15 min. The samples were then examined using fluorescence microscopy (Zeiss LSM780 confocal microscope; Zeiss, Oberkochen, Germany). Three independent measurements were performed for each sample.

### Quantification of endotoxin residue on the titanium discs (n = 6 for each group)

The remaining LPS on the titanium discs (n = 6) after different surface treatments was measured using a toxin sensor chromogenic limulus amebocyte lysate (LAL) endotoxin assay kit (GenScript, Piscataway, NJ, USA). LAL reagents were added to vials containing the treated titanium discs and an endotoxin standard. After incubation at 37 °C for 45 min, a chromogenic substrate solution (100 µL) was added and incubated at 37 °C for 6 min. A stop solution (500 µL) and color stabilizer (500 µL) were added and measured at 545 nm using a spectrophotometer.

### HGF adhesion assay (n = 6 for each group)

Gingival tissues were harvested from the maxillary tuberosity of healthy human donors. Ethical approval and informed consent were obtained from all volunteers. All experimental protocols were carried out in accordance with relevant guidelines and regulations approved by National Taiwan University Hospital (IRB no. 202002055RIND). HGF were seeded on the surface of the treated titanium discs (n = 6) in 24-well plates at a density of 2.5 × 10^4^ cells per disc and cultured for 24, 72, and 120 h for the adhesion assay. To verify and count the cell numbers, ActinGreen^Tm^ 488 (Invitrogen) to indicate a cytoskeleton and 4′,6-diamidino-2-phenylindole (DAPI) labeled nucleic acids were used. At each time point, the titanium discs were washed with PBS to remove non-attached cells and fixed in 4% paraformaldehyde for 10 min followed by ActinGreen^Tm^ 488 and DAPI. Three fields of view per sample were captured with a Zeiss LSM780 confocal microscope (Zeiss). The cell density (cells/mm^2^) of HGFs was determined using ZEN Offline (Zeiss). Three independent measurements were performed for each sample.

### Statistical analysis

Measurements and statistical analysis were evaluated by a periodontist (KL Fu) who was blind to the study design. Differences between the control and experimental groups were analyzed using a one-way analysis of variance followed by Tukey’s honest significant difference test for multiple comparisons using IBM SPSS Statistics software 23 (SPSS Inc., Chicago, IL, USA). All data are presented as means ± standard deviation. Results with *p*-values < 0.05 were considered to be significant.

## Supplementary Information

Supplementary Information.

## Data Availability

The data used to support the findings of this study are available from the corresponding author upon request.
