# Supplementary material for: Er:YAG laser irradiation enhances bacterial and lipopolysaccharide clearance and human gingival fibroblast adhesion on titanium discs
Source: Sci Rep. 2021 Dec 14;11:23954. doi: 10.1038/s41598-021-03434-1 (PMC8671504; doi:10.1038/s41598-021-03434-1)
Supplement: Supplementary file 1 — Supplementary Information. [file 41598_2021_3434_MOESM1_ESM.pdf]

Title:

Er:YAG laser irradiation enhances bacterial and lipopolysaccharide clearance and human gingival fibroblast adhesion on titanium discs

Chen-Ying Wang [1,2#], Bor-Shiunn Lee [1,3#], Ya-Ting Jhang [1,4], Kevin Sheng-Kai Ma [5], Chen-Pang Huang [1,4], Kuan-Lun Fu [1,4], Chern-Hsiung Lai [6], Wan-Yu Tseng [1,2], Mark Yen-Ping Kuo [1,2], Yi-Wen Chen [1,4*].

[*] Corresponding author

[#] Prof. Wang and Prof. Lee contributed equally to this work as first authors.

[1] Department of Dentistry, National Taiwan University Hospital, Taipei, Taiwan

[2] School of Dentistry, National Taiwan University, Taipei, Taiwan

[3] Graduate Institute of Oral Biology, School of Dentistry, National Taiwan University, Taipei, Taiwan

[4] Graduate Institute of Clinical Dentistry, School of Dentistry, National Taiwan University, Taipei, Taiwan

[5] Department of Life Science, National Taiwan University, Taipei, Taiwan

[6] College of Life Science, Kaohsiung Medical University, Kaohsiung, Taiwan

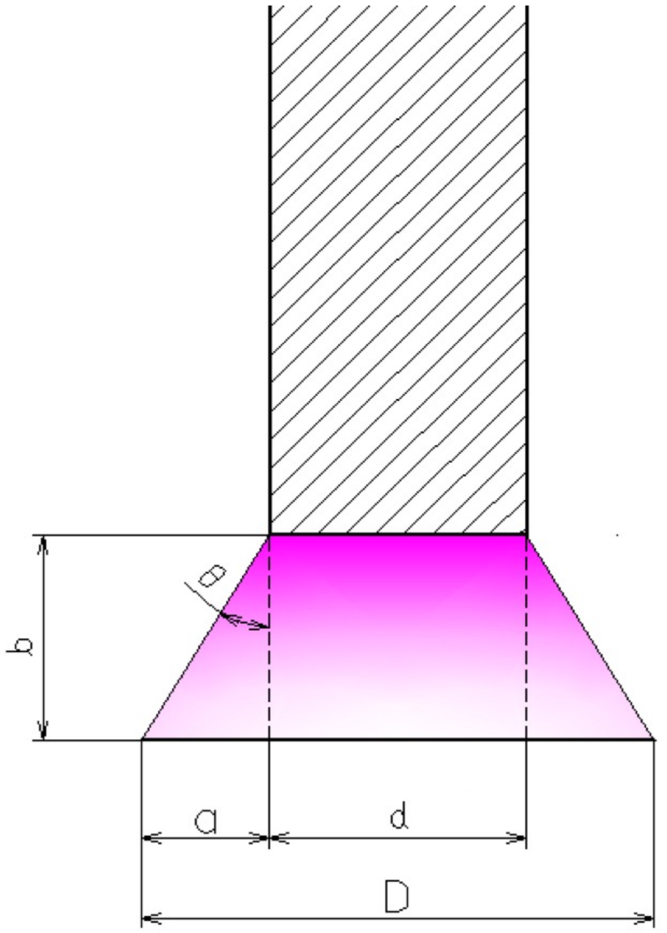

b= distance from tip end
d= diameter of the fiber core
θ= beam divergence
D= diameter of the fiber varied by distance b
S= Irradiation area

$D=d+2a=d+2b\tan\theta$
$S=\pi \times D^2/4= \pi \times (d+2b\tan\theta)^2/4$
Radiant exposure = Radiant energy x Tip transmittance/ Irradiation area(S)

b=0 mm, d=0.6mm, θ=5.2, D=0.78 mm, S=0.28 mm^2
Radiant exposure = 80mJ X 0.62 / 0.28mm^2 =177.14 mJ/mm^2 =17.714 J/cm^2

In our study:
b=1 mm, d=0.6mm, θ=5.2, D=0.78 mm, S=0.48 mm^2
Radiant exposure = 80mJ x 0.62 / 0.48mm^2 =103.33 mJ/mm^2 =10.333 J/cm^2

The declined rate is 10.333 / 17.714=0.5833

Considering the titanium surface (7.5 mm x 7.5 mm x 3.14=176.625 mm^2) and irradiation time (600 seconds)
The total radiant exposure is
80 mJ/pulse x 0.62 x 25 pulse/sec x 600 sec x 0.5833 / 176.625 mm2= 2457 mJ/mm^2=245.7 J/cm^2
